# Synthesis and Photovoltaic Effect of Electron-Withdrawing Units for Low Band Gap Conjugated Polymers Bearing Bi(thienylenevinylene) Side Chains

**DOI:** 10.3390/polym11091461

**Published:** 2019-09-06

**Authors:** Jianfeng Li, Yufei Wang, Ningning Wang, Zezhou Liang, Xu Wang, Yichun Peng, Junfeng Tong, Chunyan Yang, Yangjun Xia

**Affiliations:** 1 School of Materials and Engineering, Lanzhou Jiaotong University, Lanzhou 730070, China (Y.W.) (N.W.) (Z.L.) (X.W.) (J.T.) (C.Y.); 2 CAS Key Laboratory of Bio-Based Materials, Qingdao Institute of Bioenergy and Bioprocess Technology, Chinese Academy of Sciences, Qingdao 266101, China; 3 School of Civil Engineering, Lanzhou Institute of Technology, Lanzhou 730070, China

**Keywords:** polymer solar cells, low band gap, bi(thienylenevinylene), benzo[1,2-b:4,5-b’]dithiophene, photovoltaic property

## Abstract

A novel (*E*)-5-(2-(5-alkylthiothiophen-2-yl)vinyl)thien-2-yl (TVT)-comprising benzo[1,2-b:4,5-b’]dithiophene (BDT) derivative (BDT-TVT) was designed and synthetized to compose two donor-acceptor (D-A) typed copolymers (PBDT-TVT-ID and PBDT-TVT-DTNT) with the electron-withdrawing unit isoindigo (ID) and naphtho[1,2-c:5,6-c′]bis[1,2,5]thiadiazole (NT), respectively. PBDT-TVT-ID and PBDT-TVT-DTNT showed good thermal stability (360 °C), an absorption spectrum from 300 nm to 760 nm and a relatively low lying energy level of Highest Occupied Molecular Orbital (*E*_HOMO_) (−5.36 to –5.45 eV), which could obtain a large open-circuit voltage (*V*_oc_) from photovoltaic devices with PBDT-TVT-ID or PBDT-TVT-DTNT. The photovoltaic devices with ITO/PFN/polymers: PC_71_BM/MoO_3_/Ag structure were assembled and exhibited a good photovoltaic performance with a power conversion efficiency (PCE) of 4.09% (PBDT-TVT-ID) and 5.44% (PBDT-TVT-DTNT), respectively. The best PCE of a PBDT-TVT-DTNT/PC_71_BM-based device mainly originated from its wider absorption, higher hole mobility and favorable photoactive layer morphology.

## 1. Introduction

Polymer solar cells (PSCs) have laid special interest owing to promising qualities such as manual flexibility, being light weight, and having the potential of a large-area device prepared and developed with low-cost solution processing [1,2,3,4]. To date, the PSCs using copolymers have been researched extensively and their power conversion efficiencies (PCEs) has exceeded 14%, resulting from the development of donor-acceptor (D-A) copolymers [5,6]. However, the performances and stabilities of the PSCs still needed to be elevated to meet the practical application requirements. Among the various approaches to improve the performance and durability of PSCs, implementation of new electron donor/acceptor materials and exploration of new device architectures are required to further boost the performance and stability of PSCs. For ideal donor or acceptor materials, the energy levels arrangement, carrier mobility and absorption spectrum are important, which is correlated with the photovoltaic performance.

Due to the large planar structure of the electron-donating group benzo [1,2-b:4,5-b’] dithiophene (BDT), it has received extensive attention in constructing a D-A polymer of high performance PSCs [7,8,9,10,11,12,13,14,15,16]. Applying two-dimensional (2D)-conjugated BDT units (alkylthienyl- and alkylthio-thienyl-substituted BDT) and multifarious chemical moieties (para-alkylphenyl, metaalkoxylphenyl and 2-alkyl-3-fluorothienyl) play an important role in boosting the performance of the material and photovoltaic device, such as the adjustments of the absorption characteristic and energy levels, backbone conformation, charge transport performance, etc. [17,18,19,20,21,22,23]. Thienylenevinylene (TVT) was a strong electron donating functional group owing to the presence of an electron-rich vinyl linkage. TVT-based materials exhibited a large charge mobility due to the high planarity of the vinylidene (double bond) groups between the two thiophene units [24,25,26]. Two polymers containing an alkyl-substituted TV unit between 3-dodecylthiophenes were synthesized by Kim et al., and had a charge mobility of more than 1 cm^2^ V^−1^ s^−1^ [27]. Hou et al. introduced the TVT functional group into 4- and 8-positions of the BDT group to construct a polymer, named as PBT-TVT, and an encouraging result of 8.13% was obtained from PC_71_BM as acceptor in bulk heterojunction (BHJ) devices. Due to the prolonged conjugation of the BDT conjugated side groups, the absorption characteristic was improved distinctly and resulted in a stronger interchain π−π interaction, thus a larger hole mobility was obtained [28]. Chung et al. found that the absorption characteristic, charge mobility, energy levels and photovoltaic performance of PBDTVT-DTTPD was affected by TVT side chain groups [29]. The TVT conjugated group was introduced into the BDT-based A-D-A by Guo et al. to form BDT(TVT-SR)_2_, and the PCE of BDT(TVT-SR)_2_:IDIC photovoltaic devices reached 11.1%. Besides, the TVT-SR conjugated side chain could reduce the thermally induced phase aggregation and boost the BHJ morphological stability [30].

The electron-deficient dye Isoindigo (ID) that contained two lactam rings has a good π-conjugated planar structure and a strong electron-withdrawing ability, and thus is an ideal monomer for synthesizing a low bandgap conjugated polymer for PSCs [31,32]. Naphtho[1,2-c:5,6-c′]bis[2–4] thiadiazole (NT), a receptor structure comprised of two BT fused heterocycles, is one of the potential materials with a low band gap and high carrier mobility for solar cell receptor unit [33,34,35,36]. Because of the high π-extension structure of NT and the strong electron affinity of double heterocycles, the binding of NT and donor units to the main chain of the polymer leads to a smaller band gap (*E*_g_) and deeper Highest Occupied Molecular Orbital (HOMO) energy level. Meanwhile, the center-symmetric rigid structure of NT can enhance the interaction between NT-based polymer molecules, promote the π−π stacking effect of the polymer framework and enhance the structure order of NT-based polymer films and further improve its mobility. NT is therefore one of the potential materials with a low band gap and high carrier mobility for a solar cell receptor unit [37,38].

Considering the excellent characteristics of the above-mentioned 2D BDT-based conjugated copolymers (CPs), herein, two low band gap alternating CPs, namely PBDT-TVT-ID and PBDT-TVT-DTNT, utilizing 2D BDT-TVT as donor moiety and ID and/or 4,9-bis(4-hexylthien-2-yl)naphtho[1,2-c:5,6-c]bis[2–4]-thiadiazole (DTNT) as acceptor units, were designed and prepared. Besides, the effect on absorption characteristic, energy level, charge mobility, morphology and photovoltaic performance was researched. The resulting polymers exhibited a good thermal stability, a broad absorption spectrum (300–760 nm) and a deep HOMO energy level (−5.3 to −5.6 eV). The PSCs using PBDT-TVT-ID or PBDT-TVT-DTNT as donor and [7,7]-phenyl C_71_-butyric acid methyl ester (PC_71_BM) as acceptor obtained a PCE of 4.09% and 5.44%, respectively. The PCE based on the PBDB-TVT-ID photovoltaic device was slightly lower than that found by Ma et al., but achieved a higher *V*_oc_ (0.91 V) [30]. Compared to Chung et al., the device based on PBDB-TVT-DTNT obtained a small PCE but a larger *J*_sc_ (12.21 mA cm^−2^) [32].

## 2. Materials and Methods

### 2.1. Materials and Characteriazation

All reagents were obtained from TCI Chemical Co., Acros and Aldrich. All the solvents were further purified using a flow of nitrogen. Bistin 2,6-bis(trimethyltin)-4,8-bis[5-((*E*)-2-(4,5-di-decylthiophen-2-yl)vinyl)thien-2-yl]benzo[1,2-b:4,5-b’]dithiophene (BDT-TVTSn), dibromide *N*,*N*-di(2-butyloctyl)-6,6′-dibromoisoindigo (ID) (ID-BOBr_2_) and 4,9-bis(5-bromo-4-(2-octyl)thien-2-yl)naphtho[1,2-c:5,6-c′]bis[1,2,5]thiadiazole (DTNT-C8Br_2_) were obtained on the basis of the reported references [38,39], and related chemical structures were determined by NMR test (Figures S4–S7).

^1^H NMR and ^13^C NMR spectra were recorded on a Bruker DRX 400 spectrometer (Rheinstetten, Germany) operating at 400 MHz and was referred to tetramethylsilane (TMS). Splitting patterns were designed as *s* (singlet), *d* (doublet), *t* (triplet), *m* (multiplet), and *br*(broaden). Melting points were measured by the use of a microscopic melting point apparatus (Beijing Taike, Beijing, China), and the thermometer was uncorrected, and TGA was conducted on a TGA 2050 thermal analysis system (New Castle, DE, USA) under a heating rate of 10 °C min^−1^ and a N_2_ flow rate of 20 mL min^−1^. The average molecular weights of PBDT-TVT-ID and PBDT-TVT-DTNT were determined by gel permeation chromatography (GPC) using a polystyrene standard in a tetrahydrofuran (THF) eluting solvent. Elemental analyses were performed on a Vario EL Elemental Analysis Instrument (Elementar Co.). UV-Vis absorption spectra were measured on a UV-1800 spectrophotometer (Shimadzu, Kyoto, Japan). Cyclic voltammetry (CV) was measured on a CHI600D electro-chemical workstation (Shanghai Chenhua, Shanghai, China) at a scan rate of 100 mV s^−1^ with a nitrogen-saturated solution of 0.1 M tetrabutylammonium hexafluorophosphate (Bu_4_NPF_6_) in CH_3_CN solution. A three-electrode cell was used in all experimental, wherein polymer coated glassy carbon electrode, platinum wire and Ag/AgNO_3_ (0.01 M of AgNO_3_ in CH_3_CN) electrode were employed as the working electrode, counter electrode and reference electrode, respectively. The reference electrode was calibrated using a ferrocene/ferrocenium redox couple as an external standard, whose oxidation potential is set at −4.80 eV with respect to zero vacuum level. Polymer thin films were prepared by dropcasting 1 μL polymer chloroform solution with the concentration of 1 mg mL^−1^ onto the working electrode, and then dried in the air. The surface roughness and morphology of the thin films were characterized by atomic force microscopy on an MFP-3D-SA (Asylum Research, Santa Barbara, CA, USA) in tapping mode.^1^H NMR spectra, thermal gravimetric analysis (TGA), UV-visible absorption spectra, cyclic voltammetry (CV), transmission electron microscopy (TEM) images measurement and atomic force microscopy (AFM) were done the same as per our previous work [35,40].

### 2.2. Preparation of Photovoltaic Devices

The PSC preparation processes with a ITO/PFN/polymer:PC_71_BM/MoO_3_/Ag structure was according to Reference [35]. Noted that the active layer polymer:PC_71_BM (w/w; 1:1, 1:1.5 and 1:2) were dissolved in a chlorobenzene (CB) solution with 0.5% diphenyl sulfide (DPS) as solvent additives. A detailed description is provided in the Supporting Information.

### 2.3. Synthesis of PBDT-TVT-ID and PBDT-TVT-DTNT

#### 2.3.1. Synthesis of PBDT-TVT-ID

BDT-TVTSn (163.8 mg, 0.11 mmol) and ID-BOBr_2_ (83.3 mg, 0.11 mmol) were dissolved in a mixed solvent of freshly distilled toluene (6 mL) and anhydrous *N*,*N*-dimethylformamide (DMF) (0.7 mL). The mixture was purged with argon for 10 min, and then Pd_2_(dba)_3_ (1.5 mg) and P(*o*-tol)_3_ (3.0 mg) were added into the flask as a catalyst. After being purged for another 20 min, the reaction mixture was heated to 105 °C for 48 h under an argon atmosphere. At the end of polymerization, the polymer was end-capped with 2-tributylstannylthiophene and 2-bromo-thiophene to remove bromo and trimethystannyl end groups. The mixture was then poured into methanol. The precipitated material was collected and extracted with ethanol, acetone, hexane and toluene in a Soxhlet extractor. The solution of the copolymer in toluene was condensed to 8 mL and then poured into methanol (200 mL). The precipitation was collected and dried under vacuum overnight as a greenish-black solid (yield = 65.2%) (^1^H NMR (500 MHz, CDCl_3_), *δ* (ppm), 9.02 (*br*, ArH), 7.60−6.3 (*m*, ArH and CH_2_=CH_2_), 3.70 (*t*, CH_2_ directly linked to N), 2.75 (*br*, CH_2_, directly linked to thiophene), 2.47 (*br*, CH_2_, directly linked to thiophene), 1.80−0.80 (*m*, CH, CH_2_ and CH_3_) (Figure S8)). Elemental analysis calculated for C_110_H_152_N_2_O_2_S_6_: C, 76.51%; H, 8.87%; N, 1.62%. Found C, 76.40%; H, 8.69%; and N, 1.68%. *M*_n_ = 13.9 KDa with polydispersity index (PDI) of 1.69.

#### 2.3.2. Synthesis of PBDT-TVT-DTNT

BDT-TVTSn (163.8 mg, 0.11 mmol), DTNT-C8Br_2_ (87.0 mg, 0.11 mmol), Pd_2_(dba)_3_ (1.5 mg) and P(o-tol)_3_ (3.0 mg) were used to synthesize PBDT-TVT-DTNT. The other experimental processes were just as the preparation of the polymer PBDT-TVT-ID described above. The target polymer PBDT-TVT-DTNT was obtained as greenish-black solid (yield = 70.3%) (^1^H NMR (500 MHz, CDCl_3_), *δ* (ppm), 9.03 (*br*, ArH), 8.14 (*br*, ArH), 7.80−6.60 (*m*, ArH and CH_2_=CH_2_), 3.00−2.40 (*t*, CH_2_ directly linked to N and thiophene), 1.80−0.80 (*m*, CH_2_ and CH_3_) (Figure S9)). Elemental analysis calculated for C_105_H_135_N_4_S_10_:C, 71.09%; H, 7.67%; N, 3.16%. Found, C, 71.00%; H, 7.60%; N, 3.25%. *M*_n_ = 22.1 KDa with PDI of 2.34.

## 3. Results

### 3.1. Synthesis and Characterization

**Scheme 1:**
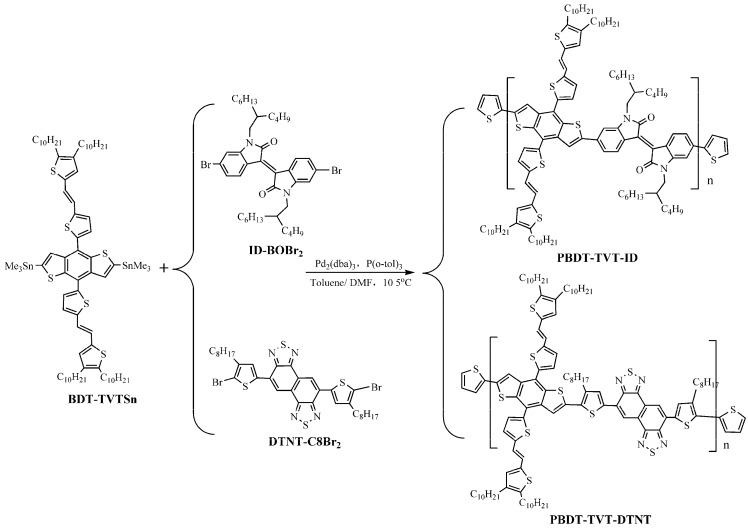
Synthetic routes for the polymers PBDT-TVT-ID and PBDT-TVT-DTNT.

The synthetic routes of PBDT-TVT-ID and PBDT-TVT-DTNT are outlined in Scheme 1. The resultant copolymers were prepared by Stille-coupling polymerization with distannyl BDT-TVTSn and dibromide monomers ID-BOBr_2_/DTNT-C_8_Br_2_ using Pd_2_(dba)_3_ and P(*o*-tol)_3_ as a catalyst. PBDT-TVT-DTNT is soluble in a normal organic solvent such as toluene, chlorobenzene and *o*-dichlorobenzene. However, the solubility of PBDB-TVT-ID is not as good as that of PBDB-TVT-DTNT, but they all dissolve well in chlorobenzene.

**Figure 1:**
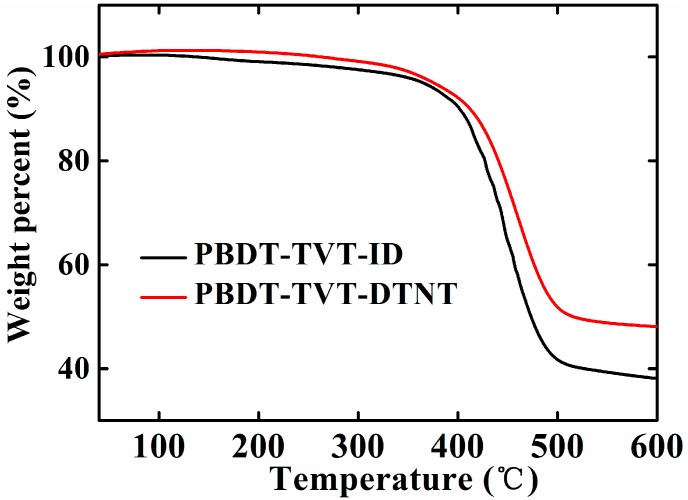
Thermal gravimetric analysis (TGA) characteristics of PBDT-TVT-ID and PBDT-TVT-DTNT.

TGA characteristics of the PBDT-TVT-ID and PBDT-TVT-DTNT were displayed in Figure 1. The TGA measurement indicated that the two polymers showed good thermal stability and the decomposition temperatures (5% weight loss) of PBDT-TVT-ID and PBDT-TVT-DTNT were 365 and 376 °C, respectively. Besides, differential scanning calorimetry (DSC) measurements (shown in Figure S10) of the two polymers found no significant thermal transitions, which imply the two polymers have an amorphous state.

### 3.2. Optical and Electrochemical Performance

**Figure 2:**
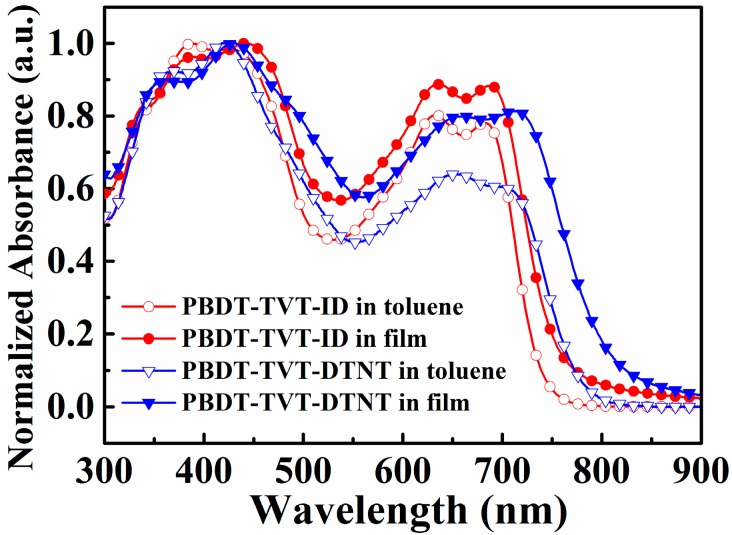
Normalized UV-visible absorption spectra of the polymers in toluene solution and thin films.

The absorption characteristic curves of PBDT-TVT-ID and PBDT-TVT-DTNT in toluene solution and thin films were exhibited in Figure 2. The related calculation results are listed in Table 1. The absorption range of PBDT-TVT-ID was from 300 to 743 nm and the maximum absorption peak (*λ*_max_) located in 687 nm. However, PBDT-TVT-DTNT exhibited a broader spectrum than PBDT-TVT-ID and red-shifted to a long wavelength region of 780 nm, which was attributed to the produced stronger ICT interaction between bilateral thiophene and the NT unit as result of introducing DTNT [41]. Compared to the solution state, the absorption curves of PBDT-TVT-ID and PBDT-TVT-DTNT film hardly changed, but the absorption edges were broadened to 760 nm and 817 nm, respectively [42]. Particularly, PBDT-TVT-DTNT-based film showed a notable increase in intramolecular charge transfer (ICT) absorption band than PBDT-TVT-ID, suggesting PBDT-TVT-DTNT having a stronger aggregation tendency and more ordered microstructure in the solid film. According to *E*_g_ = 1240/*λ*_onset_, the optical bandgaps (*E*_g_) of the PBDT-TVT-ID and PBDT-TVT-DTNT obtained from film absorption edges were 1.63 and 1.52 eV, respectively.

**Figure 3:**
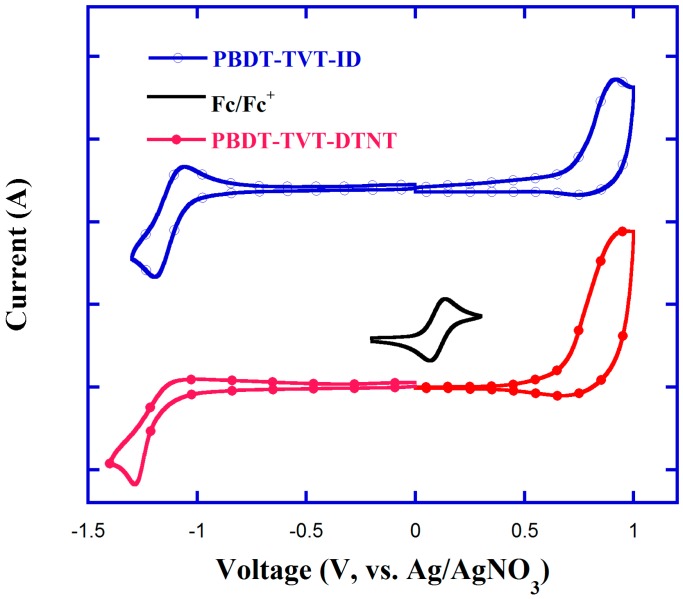
CV curves of PBDT-TVT-ID and PBDT-TVT-DTNT.

The HOMO energy level (*E*_HOMO_) of the polymer could be determined by CV testing and the related condition was the same as in Reference [31]. From Figure 3, the onset potentials of PBDT-TVT-ID and PBDT-TVT-DTNT were located in 0.72/−1.02 V and 0.65/−1.11 V, respectively. The corresponding *E*_HOMO_/*E*_LUMO_ levels were calculated to be −5.42/−3.68 eV and −5.35/−3.59 eV using a formula *E*_HOMO_ = −e(*φ*_ox_ + 4.70) (eV) and *E*_LUMO_ = −e(*φ*_red_ + 4.70) (eV), respectively (Table 1). Remarkably, the *E*_HOMO_ of PBDT-TVT-ID is deeper than the *E*_HOMO_ of PBDT-TVT-DTNT, and a higher *V*_OC_ can be achieved in a PBDT-TVT-ID-based PSC.

### 3.3. Hole Mobility

**Figure 4:**
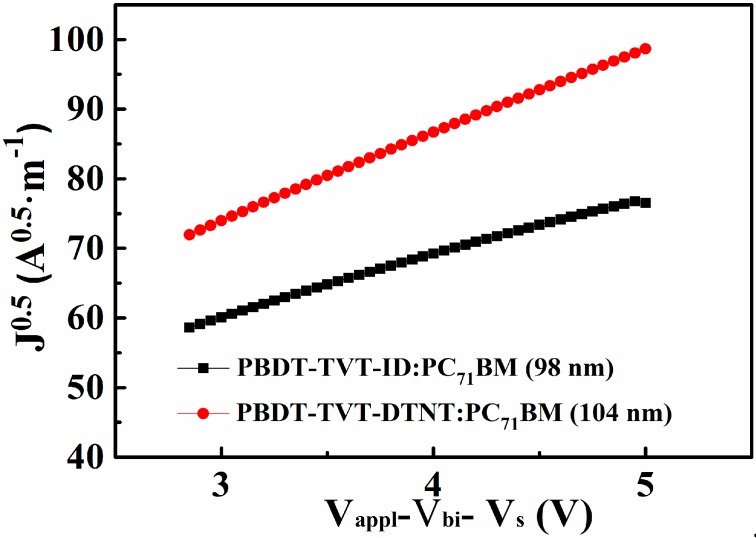
*J*-*V* characteristics of hole-only devices.

To determine the influence of the polymer on charge transport, the hole-only devices with the structure ITO/PEDOT:PSS/polymer:PC_71_BM/MoO_3_/Ag (using the best polymer:PC_71_BM ratio) were prepared. The dark *J*-*V* curves of PBDT-TVT-ID and PBDT-TVT-DTNT are displayed in Figure 4, and the thicknesses were 98 nm and 104 nm for PBDT-TVT-ID:PC_71_BM and PBDT-TVT-DTNT:PC_71_BM blend films, respectively. The hole mobilities of the polymers could be estimated by the equation [43,44,45]:

(1) $$J=\frac{9}{8}\varepsilon_{0}\varepsilon_{r}\mu \frac{V^{2}}{d^{3}}.$$

Therefore, the hole mobilities were calculated to be 2.96 × 10^−5^ cm^2^ V^−1^ s^−1^ and 6.73 × 10^−5^ cm^2^ V^−1^ s^−1^ for PBDT-TVT-ID and PBDT-TVT-DTNT, respectively. PBDT-TVT-DTNT has a higher hole mobility, which was in accordance with a higher polymer backbone coplanarity obtained from the latter density functional theory (DFT) calculation. The higher hole mobility of PBDT-TVT-DTNT facilitates the achievement of high *J*_SC_ and FF, and thus a high PCE.

### 3.4. Theoretical Calculations

**Figure 5:**
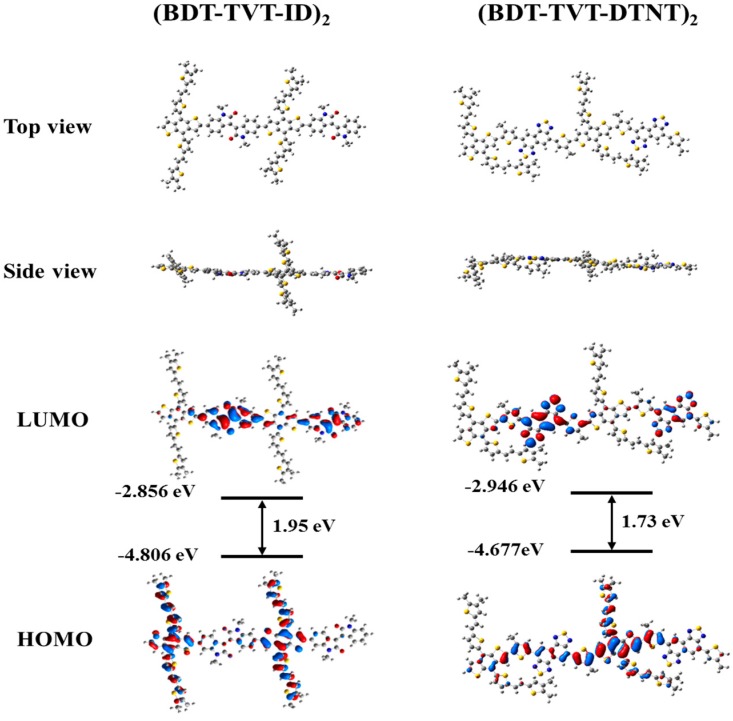
Optimized molecular geometries and molecular orbital of the Highest Occupied Molecular Orbital (HOMO) and Lowest Unoccupied Molecular Orbital (LUMO) for polymers.

To further explore the electronic properties of PBDT-TVT-ID and PBDT-TVT-DTNT, density functional theory (DFT) was used to simulate the molecular geometry and electron density state distribution of the two polymers. The DFT calculations were carried on the B3LYP-D3(BJ)/6-31G(d) level by Gaussian 09, and two repeating units as the calculation model [46,47]. Note that the long side chain (2-butyloctyl, octyl and decyl) is replaced by a methyl group to simplify the calculation. The optimized geometries and frontier orbitals (HOMO and Lowest Unoccupied Molecular Orbital (LUMO)) of PBDT-TVT-ID and PBDT-TVT-DTNT are displayed in Figure 5 and Table 1, respectively. As shown in Figure 5, the HOMO orbital of (BDT-TVT-ID)_2_ is mainly delocalized on the BDT-TVT moiety and one (BDT-TVT-DTNT)_2_ is preferentially delocalized across the conjugated main chain, whereas the LUMO orbitals of both copolymers are preferentially localized on the electron-deficient ID and/or the DTNT segment. In addition, the DFT calculated HOMO, LUMO and *E*_g_ as –4.808, –2.856 and 1.95 eV for PBDT-TVT-ID and –4.677, –2.946 and 1.73 eV for PBDT-TVT-DTNT, respectively. Remarkably, when DYNT is replaced by ID, HOMO shows a slight deepening, which is agreement with the results of the CV test. Furthermore, as shown in Figure S11, the torsional angles between the subunits (20.92°, 22.35° and 19.16°) in PBDT-TVT-ID was slightly larger than the ones (9.62°, 12.02° and 9.70°) in PBDT-TVT-DTNT; that is to say, PBDT-TVT-DTNT showed a higher coplanarity which was beneficial for enhancing the backbone π–π stacking in the solid state and charge mobility, as well as having a higher photovoltaic performance.

### 3.5. Photovoltaic Performance

**Figure 6:**
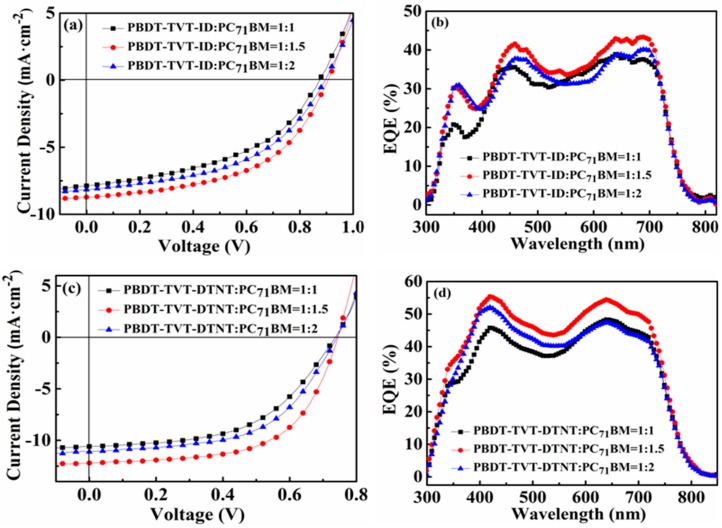
(**a**,**c**) *J*-*V* curves of the PSCs based on PBDT-TVT-ID and PBDT-TVT-DTNT; (**b**,**d**) EQE spectrums of the related PSCs.

To have insight into the photovoltaic performance of PBDT-TVT-ID and PBDT-TVT-DTNT, the PSCs device with ITO/PFN/active layers/MoO_3_/Ag were assembled. The photoactive layers using an electron donor (PBDT-TVT-ID or PBDT-TVT-DTNT) and an electron acceptor (PC_71_BM) were dissolved in CB. The weight ratios of copolymers were: PC_71_BM of 1:1 to 1:1.5 and then up to 1:2 with 0.5% DPS as solvent additives were configured to an initial optimization. The *J-V* characteristics and related calculation result are shown in Figure 6 and Table 2, respectively. Notably, the photovoltaic devices based on PBDT-TVT-ID showed the best PCE (4.09%), with a *V*_OC_ of 0.91 V, a *J*_SC_ of 8.72 mA cm^−2^ and an *FF* of 51.49%, while the devices with PBDT-TVT-DTNT obtained a larger PCE of 5.44%, with a *V*_OC_ of 0.74 V, a *J*_SC_ of 12.21 mA cm^−2^ and an FF of 60.26% mA·cm^−2^, as well as an *FF* of 45.8%. Note that the PBDT-TVT-ID photovoltaic devices exhibited a higher *V*_OC_ (0.91 V) than the PBDT-TVT-DTNT-based device. Regardless of the effect of the recombination process and shunt resistance, the difference between the polymer HOMO and PC_71_BM LUMO plays an important role in affecting *V*_OC_. Compared to PBDT-TVT-DTNT, the deeper HOMO energy level of PBDT-TVT-ID can achieve a higher *V*_OC_ in PSCs. But, the devices with PBDT-TVT-ID exhibited a poorer *FF* of 51.49%, which resulted from the rougher surface morphology and larger phase separation of PBDT-TVT-ID. Meanwhile, the PSC of PBDT-TVT-DTNT possess a broader spectral response range and can capture more photons, which may be considered as an important factor to enhance the *J*_SC_ of PBDT-TVT-DTNT.

### 3.6. Morphology Study

**Figure 7:**
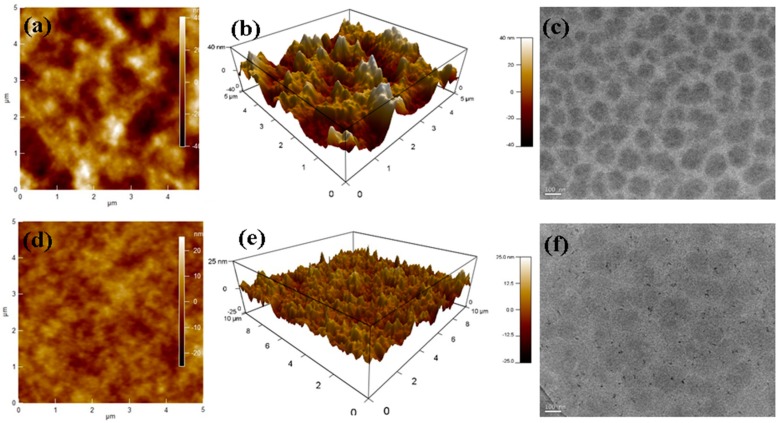
AFM and TEM images of polymer/PC_71_BM blend films: (**a**,**d**) AFM height; (**b**,**e**) 3D height; (**c**,**f**) TEM; (**a**–**c**) PBDT-TVT-ID; and (**d**–**f**) PBDT-TVT-DTNT.

The performances of BHJ PSCs are strongly associated with the morphologies of their active layer [40,48]. Therefore, the real space morphologies on the surface and in the bulk of the photoactive layer films under the best ratio conditions were researched by AFM and TEM. The root-mean-square (RMS) roughness values obtained from the height image were 13.5 nm and 10.3 nm for the blends of PBDT-TVT-ID and PBDT-TVT-DTNT, respectively (Figure 7). It can be seen that there is a serious phase separation between PBDT-TVT-ID and PC_71_BM. Meanwhile, from the TEM, some PC_71_BM exhibit severe aggregations over 150 nm, indicating that a good interpenetrating network structure cannot be formed. Under the above circumstances, photogenerated excitons are unable to achieve effective dissociation and charge collection in the PSC, resulting in loss of photocurrent. This explains why the devices based on PBDT-TVT-ID have a low FF and *J*_SC_. In contrast, the PBDT-TVT-DTNT:PC_71_BM blended film exhibits a better bicontinuous interpenetrating network and smooth interface, which can boost charge transport, and thus a larger FF and *J*_SC_ are obtained.

## 4. Conclusions

Two new solution-processed low band gap copolymers, named PBDT-TVT-ID and PBDT-TVT-DTNT, were designed and synthesized by copolymerizing between bistin BDT-TVTSn and dibromide ID-BOBr_2_ and/or DTNT-C8Br_2_, respectively. Both polymers exhibited good thermal stability, broad absorption spectra and relatively low HOMO energy levels. The device with PBDT-TVT-ID/PC_71_BM and PBDT-TVT-DTNT/PC_71_BM exhibited good photovoltaic performance with PCEs of 4.09% and 5.44%, respectively. The results demonstrated that a BDT unit containing TVT conjugated side chains may be a promising electron-donor building block for high performance solution-processed PSCs. The device efficiency based on this kind of polymer can be further improved by controlling the film morphology and energy level of the polymer by side chain engineering and a fluorination strategy.

## Figures and Tables

**Table 1:** Absorption characteristic and cyclic voltammetry (CV) parameters.

Polymer	λ_onset_ (nm)	*E*_g_^1^ (eV)	*E*_g_^elect^	HOMO ^2^ (eV)	LUMO ^3^ (eV)	HOMO ^4^ (eV)	LUMO ^4^ (eV)
PBDT-TVT-ID	760	1.63	1.74	−5.42	−3.68	−4.81	−2.86
PBDT-TVT-DTNT	817	1.52	1.76	−5.35	−3.59	−4.68	−2.95

^1^ Calculated from the empirical equation: *E*_g_ = 1240/λ_onset_; ^2^ *E*_HOMO_ = −e (*φ*_ox_ + 4.70) (eV); ^3^ *E*_LUMO_ = −e (*φ*_red_ + 4.70) (eV); ^4^ DFT data calculated from dimer model compound.

**Table 2:** The photovoltaic parameters of the polymer solar cells (PSCs) with PBDT-TVT-ID or PBDT-TVT-DTNT.

Active Layer	Additive	*V*_OC_ (V)	*J*_SC_ (mA cm^−2^)	FF (%)	PCE (%) Best	PCE (%) Average ^a^
PBDT-TVT-ID:PC_71_BM=1:1	0.5% DPS	0.90	7.82	44.60	3.16	3.10
PBDT-TVT-ID:PC_71_BM=1:1.5	0.5% DPS	0.91	8.72	51.49	4.09	3.89
PBDT-TVT-ID:PC_71_BM=1:2	0.5% DPS	0.90	8.16	48.38	3.55	3.42
PBDT-TVT-DTNT:PC_71_BM=1:1	0.5% DPS	0.74	10.60	52.00	4.08	3.91
PBDT-TVT-DTNT:PC_71_BM=1:1.5	0.5% DPS	0.74	12.21	60.25	5.44	5.26
PBDT-TVT-DTNT:PC_71_BM=1:2.	0.5% DPS	0.74	11.09	55.11	4.53	4.34

^a^ More than 15 solar cell samples were evaluated.

## Supplementary Materials

The following are available online at https://www.mdpi.com/2073-4360/11/9/1461/s1, Figure S1 ^1^H-NMR spectrum of Compound 1 in CDCl_3_ solution; Figure S2 ^1^H-NMR spectrum of Compound 3 in CDCl_3_ solution; Figure S3 ^1^H-NMR spectrum of Compound 4 in CDCl_3_ solution; Figure S4 ^1^H-NMR spectrum of BDT-TVTSn in CDCl_3_ solution; Figure S5 ^13^C-NMR spectrum of BDT-TVTSn in CDCl_3_ solution; Figure S6 ^1^H-NMR spectrum of M2 in CDCl_3_ solution; Figure S7 ^1^H-NMR spectrum of M3 in CDCl_3_ solution; Figure S8 ^1^H-NMR spectrum of PBDT-TVT-ID; Figure S9 ^1^H-NMR spectrum of PBDT-TVT-DTNT; Figure S10 DSC scan curves of polymer; Figure S11 Dihedral angles for model compound (BDT-TVT-ID)_2_ and (BDT-TVT-DTNT)_2_.
