# Supplementary material for: Synthesis and Photovoltaic Effect of Electron-Withdrawing Units for Low Band Gap Conjugated Polymers Bearing Bi(thienylenevinylene) Side Chains
Source: Polymers (Basel). 2019 Sep 6;11(9):1461. doi: 10.3390/polym11091461 (PMC6780057; doi:10.3390/polym11091461)
Supplement: Supplementary file 1 [file polymers-11-01461-s001.pdf]

polymers

MDPI

Supplementary Information

Synthesis and Photovoltaic Effect of Electron-Withdrawing Units for Low Band Gap Conjugated Polymers Bearing Bi(thienylenevinylene) Side Chains

Jianfeng Li [1,*], Yufei Wang [1], Ningning Wang [1], Zezhou Liang [1,3], Xu Wang [1], Yichun Peng [2], Junfeng Tong [1], Chunyan Yang [1] and Yangjun Xia [1,*]

[1] School of Materials and Engineering, Lanzhou Jiaotong University, Lanzhou 730070, China; yufeiwang96@hotmail.com (Y.W.); ningningwang18@163.com (N.W.); zezhouliang@hotmail.com (Z.L.); wx2512093772@163.com (X.W.); tongjunfeng139@163.com (J.T.); YChY-5@126.com (C.Y.)
[2] School of Civil Engineering, Lanzhou Institute of Technology, Lanzhou 730070, China; pycljf@163.com
[3] CAS Key Laboratory of Bio-Based Materials, Qingdao Institute of Bioenergy and Bioprocess Technology, Chinese Academy of Sciences, Qingdao 266101, China
* Correspondence: ljfpyc@163.com (J.L.); xiayangjun2015@126.com (Y.X.); Tel.: +86-931-495-6022 (J.L. & Y.X.)

Received: 22 August 2019; Accepted: 2 September 2019; Published: date

1. Experimental section

1.1. Synthesis of the Monomers

2,3-Didecylthiophene (compound 1) was synthesized according to a previously reported method [1].

4,5-bis-decyl-thiophene-2-carbaldehyde (1). To a solution of 2,3-bis-decyl-thiophene (5.0 g, 13.7 mmol) in sym-dichloroethane (30 mL), anhydrous DMF (1.5g, 20.6 mmol) was added dropwise under nitrogen atmosphere, then phosphoryl chloride (3.16g, 20.6 mmol) was added slowly at 0 °C. The mixture was stirred at 25 °C for 20 min and then stirred under 80 °C for 12 h. After cooled to room temperature, 50 mL of water was added to quench the reaction and $NaCO_3$ was added to adjust the pH to neutral, then the mixture was extracted with dichloromethane. The organic layer was washed with water, then dried over anhy-drous $NaSO_4$. After removal of the solvent under reduced pressure, the residues was purified on silica gel chromatography using a petroleum ether/ethyl acetate (10/1, V/V) as eluent to afford product 1 (4.4 g, Yield = 81.5%).

^{1}H NMR (400 MHz, $CDCl_3$), δ (ppm): 9.75 (*s*, 1H), 7.51 (*s*, 1H), 2.77 (*t*, 2H), 2.52 (*t*, 2H), 1.62–1.69 (*m*, 4H), 1.26-1.30 (*m*, 28H), 0.88 (*t*, 6H). (Figure S1).

(E)-2-(2-(4,5-Didecylthiophene)-2-yl)vinyl)thiophene (3). Compound 1 (2.5 g, 6.36 mmol) was added to a solution of diethyl(thiophen-2-ylmethyl)phosphonate (compound 2) (1.94 g, 8.27 mmol) and sodium tert-butoxide (0.79g, 8.27 mmol) in THF (50 mL) at 0 °C. After 12 h of stirring, the reaction was quenched by water and extracted by ethyl acetate. The solvent was removed via rotary evaporation, and then the crude product was purified by silica gel column chromatography with petroleum ether as eluent. Compound 3 (2.51g, yield = 60%) was obtained as sticky yellow oil. ^{1}H NMR (400 MHz, $CDCl_3$), δ (ppm): 9.75 (*s*, 1H), 7.51 (*s*, 1H), 2.61 (*t*, 2H), 2.35 (*t*, 2H), 1.47 (*m*, 4H), 1.19–1.28 (m, 28H), 0.79 (*t*, 6H). (Figure S2).

4,8-bis[5-((E)-2-(4,5-decyl-thiophen-2-yl)vinyl))thiophen-2-yl]benzo[1,2-b:4,5-b′]dithiophene (4). n-Butyllithium (2.5 M, 1.8 mL) was added drop wise into a solution of compound 3 (1.93 g, 4.09 mmol) in THF (40 mL) at 0 °C under protection of argon, and then the mixture was warmed to 50°C and stirred for 0.5 h. Subsequently, benzo[1,2-b:4,5-b′]dithiophene-4,8-dione (0.30 g, 1.36 mmol) was injected in the flask and stirred for 1 h, and then a solution of $SnCl_2{\cdot}2H_2O$ (2.12 g, 9.41 mmol) in 10% HCl was added at room temperature and stirred for another 2 h. After the mixture was extracted by

water and ethyl acetate, the organic phase was concentrated by removing of solvent, and the raw product was purified by silica gel chromatography to get pure compound 4 as yellow solid (0.91g, yield = 57%). ^{1}H NMR ($CDCl_3$, 600MHz), δ (ppm): 7.70 (*d*, 2H), 7.50 (*d*, 2H), 7.38 (*d*, 2H), 7.12 (*d*, 2H), 7.04 (*d*, 2H), 6.97 (*d*, 2H), 6.80 (*s*, 2H), 2.71 (*t*, 4H), 2.46 (*t*, 4H), 1.65 (*m*, 8H), 1.35–1.20 (*m*, 56H), 0.89 (*t*,12H). (Figure S3).

4,8-bis[5-((E)-2-(4,5-decyl-thiophen-2-yl)vinyl))thiophen-2-yl]benzo[1,2-b:4,5-b′]dithiophene-2,6-diyl)bis-(trimethylstannane) (M1). Under protection of argon, nbutyllithium (1.6 M, 1.4mL) was added dropwise into a solution of compound 4 (0.9 g, 0.795 mmol) in THF (60 mL) at −55°C. After 0.5 h of reaction, additional nbutyllithium (1.6 M, 0.56 mmol) was added to the reaction flask. After 2 h, trimethyltin chloride (0.63 g, 3.18 mmol) solution was added and stirred for 30 min, and then the cooling bath was removed and stirred for another 4 h at ambient temperature. The mixture was poured into water and extracted by ether, and the organic layer was concentrated to obtain the crude compound M1. *Iso*-propyl alcohol was used to recrystallize for further purification, after which the pure BDT-TVTSn (0.80 g, yield = 67.6%) was obtained as orange oily viscous solid. ^{1}H NMR ($CDCl_3$, 600MHz), δ (ppm): 7.71 (*s*, 2H), 7.39 (*d*, 2H), 7.14 (*d*, 2H), 7.05 (*d*, 2H), 6.98 (*d*, 2H), 6.81 (*s*, 2H), 2.71 (*t*, 4H), 2.46 (*t*, 4H), 1.39-1.28 (*m*, 56H), 0.89 (*t*, 12H), 0.42 (*t*, 18H). (Figure S4); ^{13}C NMR ($CDCl_3$, 600MHz), δ (ppm): 143.62, 143.29, 142.83, 139.03, 138.58, 137.82, 137.33, 130.92, 128.71, 128.54, 125.80, 122.44, 122.14, 119.71, 110.44, 31.91, 31.74, 30.69, 29.64, 29.62, 29.62, 29.58, 29.53, 29.47, 29.44, 29.35, 29.32, 28.17, 28.11, 22.69, 14.12. (Figure S5). The melting point of BDT-TVT is 63–66 °C

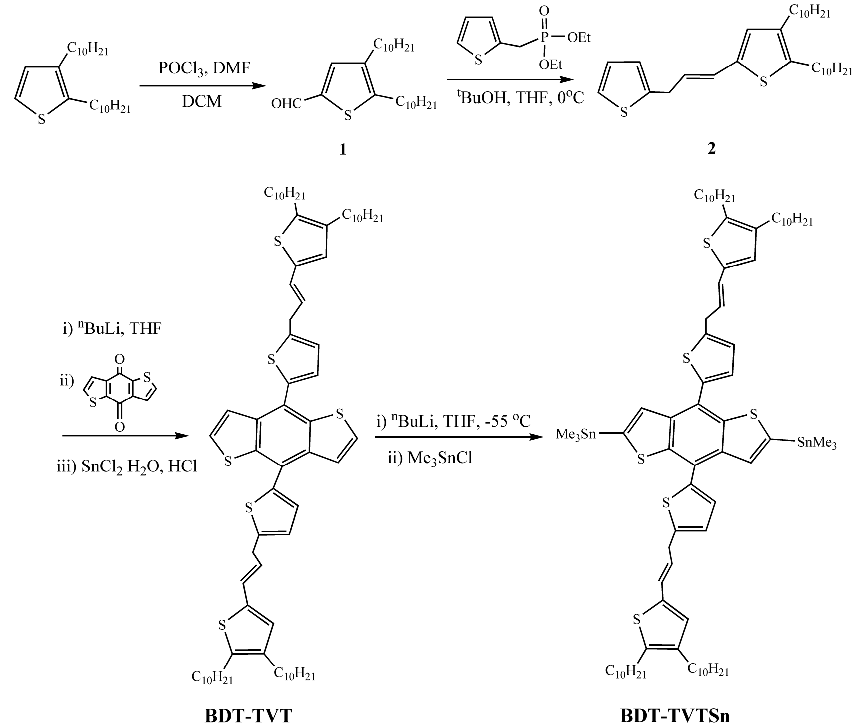

BDT-TVT **BDT-TVTSn**

Scheme S1. Molecular structure and synthetic procedures of BDT-TVTsn.

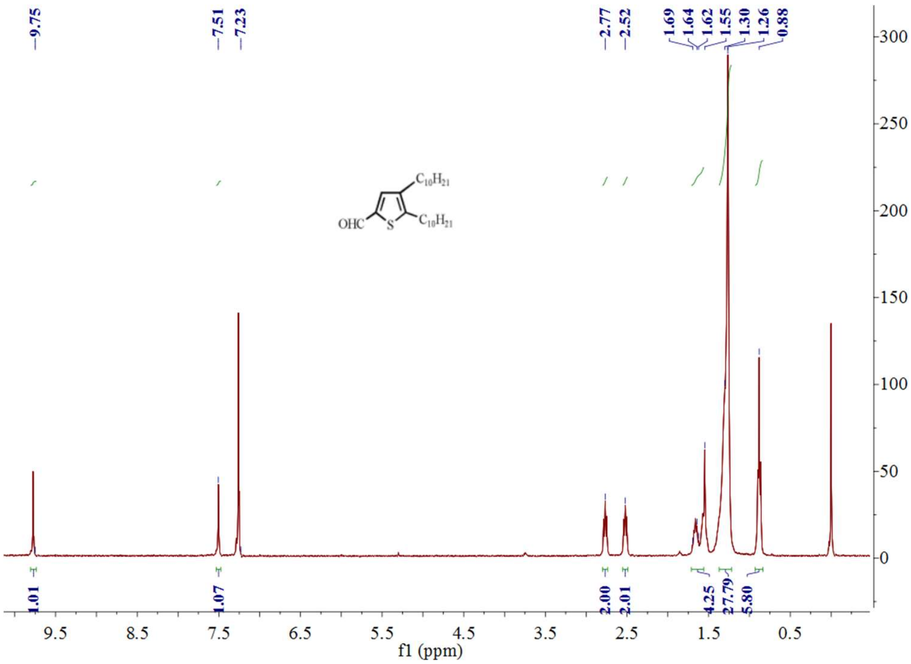

Figure S1. ^{1}H-NMR spectrum of Compound 1 in $CDCl_3$ solution.

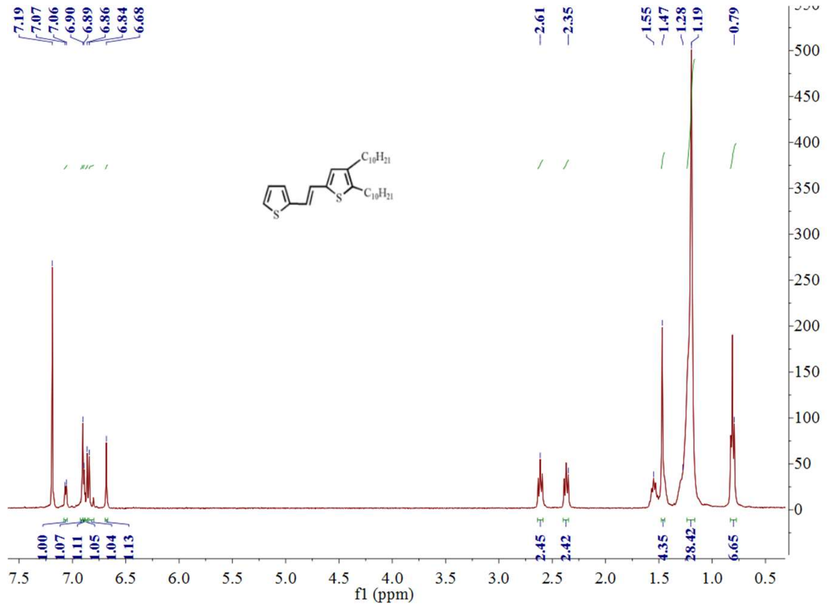

Figure S2. ^{1}H-NMR spectrum of Compound 3 in $CDCl_3$ solution.

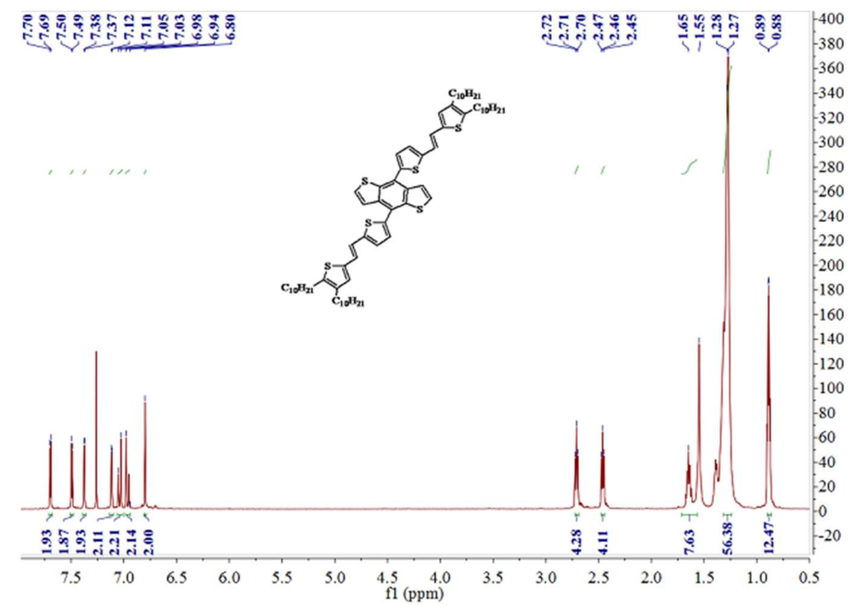

Figure S3. ^{1}H-NMR spectrum of Compound 4 in $CDCl_3$ solution

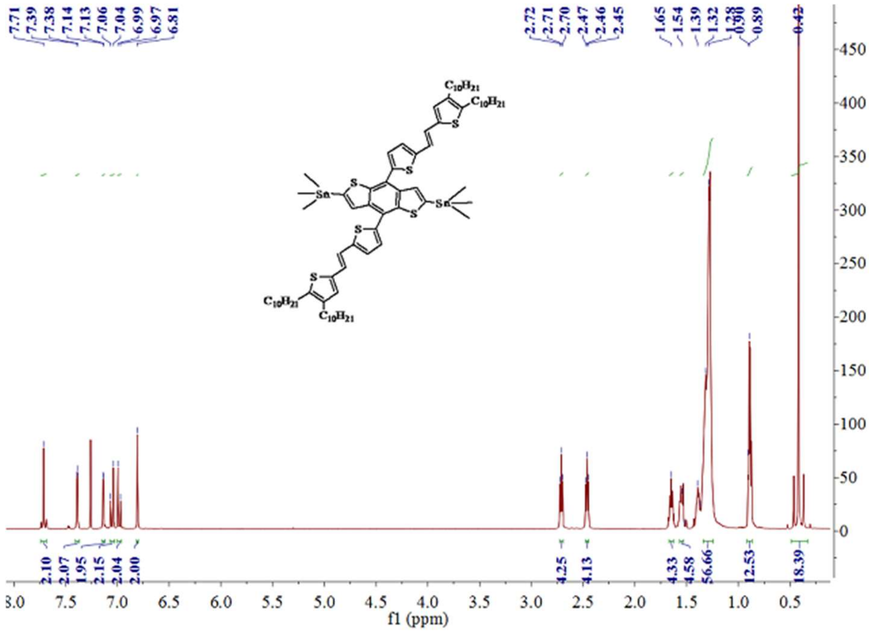

Figure S4. ^{1}H-NMR spectrum of BDT-TVTSn in $CDCl_3$ solution.

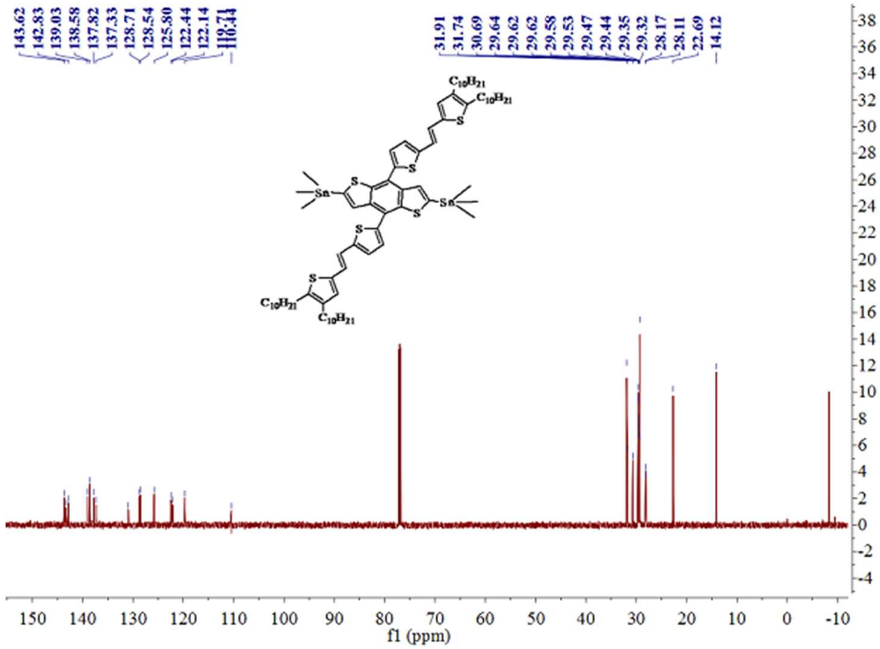

Figure S5. ^{13}C-NMR spectrum of BDT-TVTSn in $CDCl_3$ solution.

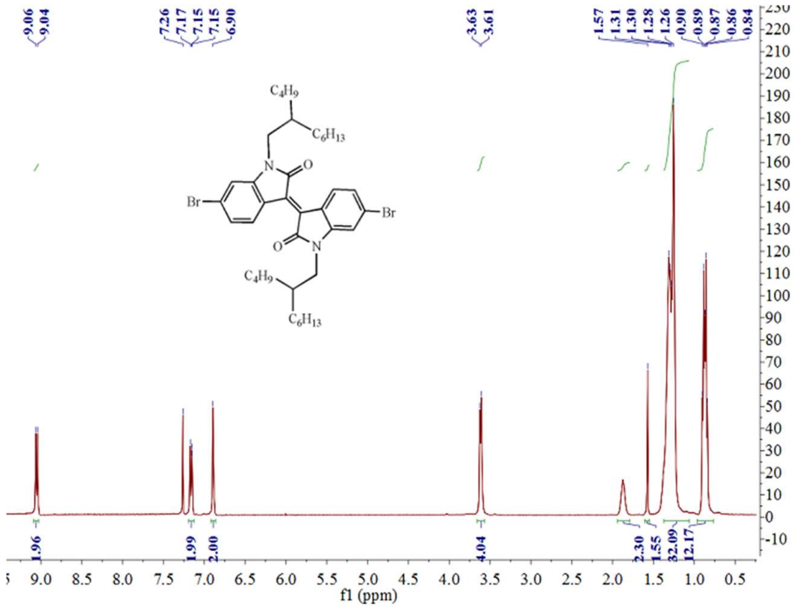

Figure S6. ^{1}H-NMR spectrum of M2 in $CDCl_3$ solution.

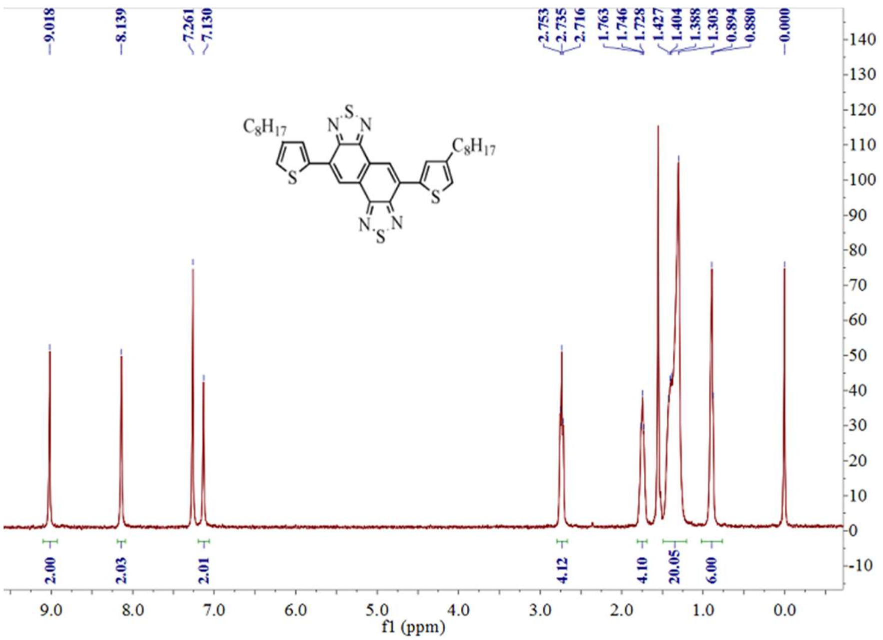

Figure S7. ^{1}H-NMR spectrum of M3 in $CDCl_3$ solution.

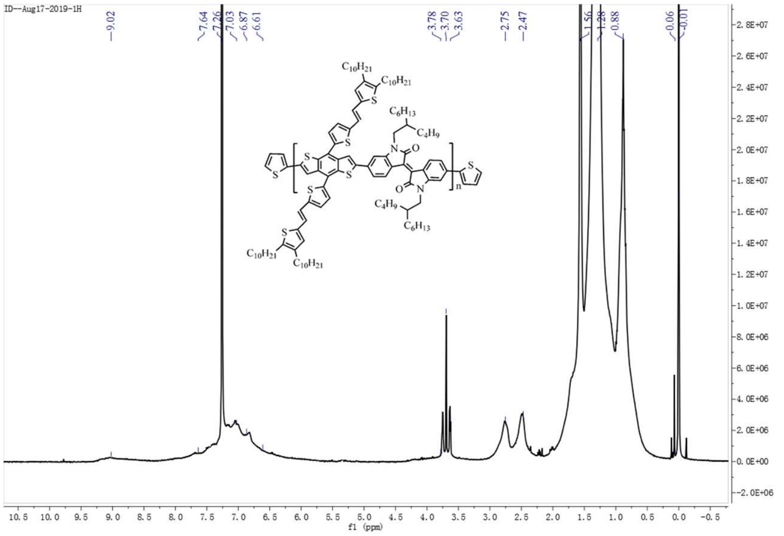

Figure S8. ^{1}H-NMR spectrum of PBDT-TVT-ID.

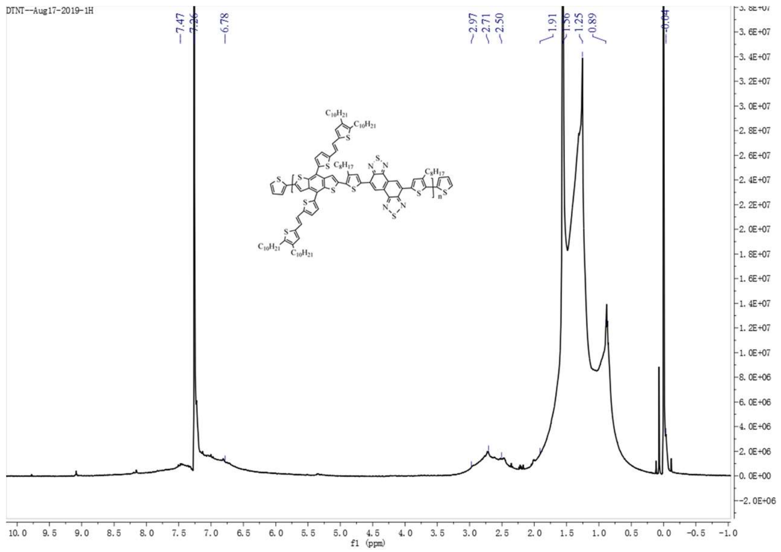

Figure S9. ^{1}H-NMR spectrum of PBDT-TVT-DTNT.

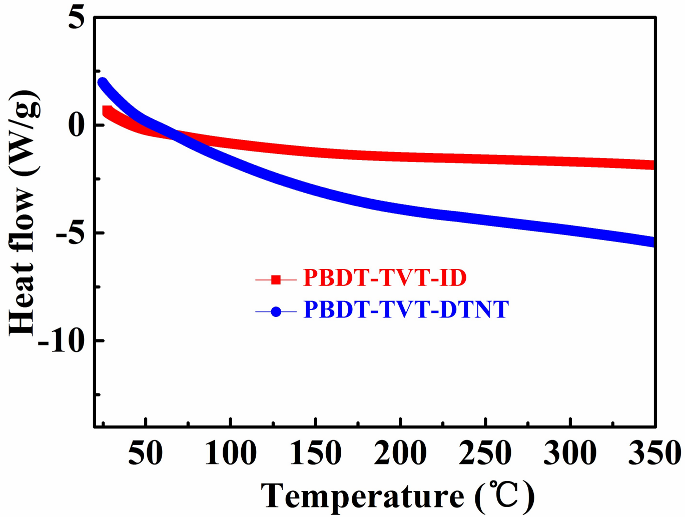

Figure S10. DSC scan curves of polymer.

1.2. Characterization

^{1}H NMR and ^{13}C NMR spectra were recorded on a Bruker DRX 400 spectrometer (Rheinstetten, Germany) operating at 400 MHz and was referred to tetramethylsilane (TMS). Splitting patterns were designed as *s* (singlet), *d* (doublet), *t* (triplet), *m* (multiplet), and *br* (broaden). Melting points were measured by the use of a microscopic melting point apparatus made by the Beijing Taike instrument limited company, and the thermometer was uncorrected, and TGA was conducted on a TGA 2050 thermal analysis system (TA instruments, New Castle, DE, USA) under a heating rate of 10 °C min^{-1} and a N_2 flow rate of 20 mL min^{-1}. The average molecular weights of PBDT-TVT-ID and PBDT-TVT-DTNT were determined by gel permeation chromatography (GPC) using a polystyrene standard in a tetrahydrofuran (THF) eluting solvent. Elemental analyses were performed on a Vario EL Elemental Analysis Instrument (Elementar Co.). UV-Vis absorption spectra were measured on a UV-1800

spectrophotometer (Shimadzu, Kyoto, Japan). Cyclic voltammetry (CV) was measured on a CHI600D electro-chemical workstation (Shanghai Chenhua, Shanghai, China) at a scan rate of 100 mV s^{-1} with a nitrogen-saturated solution of 0.1 M tetrabutylammonium hexafluorophosphate (Bu_4NPF_6) in CH_3CN solution. A three- electrode cell was used in all experimental, wherein polymer coated glassy carbon electrode, platinum wire and Ag/$AgNO_3$ (0.01 M of $AgNO_3$ in CH_3CN) electrode were employed as the working electrode, counter electrode and reference electrode, respectively. The reference electrode was calibrated using a ferrocene/ferrocenium redox couple as an external standard, whose oxidation potential is set at −4.80 eV with respect to zero vacuum level. Polymer thin films were prepared by dropcasting 1 μL polymer chloroform solution with the concentration of 1 mg mL^{-1} onto the working electrode, and then dried in the air. The surface roughness and morphology of the thin films were characterized by atomic force microscopy on an MFP-3D-SA (Asylum Research, Santa Barbara, CA, USA) in tapping mode.

1.3. Fabrication and Characterization of PSCs

A patterned indium tin oxide (ITO) coated glass with a sheet resistance of 10−15 Ω/square was sequentially cleaned by detergent, deionized water, acetone and iso-propanol in an ultrasonic cleaner. The inverted structure was ITO/PFN/active layer/MoO_3/Ag. The PFN interlayer material was dissolved in methanol in the presence of a small amount of acetic acid (2 μL mL^{-1}) and its solution (1 mg mL^{-1}) was spin-coated on the top of the precleaned ITO substrate, forming a thin interlayer with an initial thickness of 10 nm. The active layer (~100 nm) was deposited on top of the interlayer layer by spin-casting from CB solution containing the studied polymer/$PC_{71}BM$ (w/w; 1:1, 1:1.5 and 1:2), respectively. At a base pressure of 5×10^{-5} Pa, a 8 nm MoO_3 was thermally evaporated at an evaporation rate of 0.1 Å s^{-1}. Finally, a 100 nm Ag layer was evaporated with a shadow mask. The overlapping area between the cathode and anode defined a pixel size of 0.1 cm^2. The thickness values of the evaporated MoO_3 and Ag were monitored by a quartz crystal thickness/ratio monitor (SI-TM206, Shenyang Sciens Co.). All the fabrication processes were carried out inside a controlled atmosphere in a nitrogen drybox (Etelux Co.) containing less than 1 ppm oxygen and moisture. The PCEs of the resulting PSCs were measured under 1 sun, AM 1.5 G (Air mass 1.5 global) condition using a solar simulator (XES-70S1, San-EI Electric Co.) with irradiation of 100 mW cm^{-2}. The current density-voltage (*J-V*) characteristics were recorded with a Keithley 2400 source-measurement unit. The spectral responses of the devices were measured with a commercial external quantum efficiency (EQE)/incident photon to charge carrier efficiency (IPCE) setup (7-SCSpecIII, Beijing 7-star Opt. In. Co.) A calibrated silicon detector was used to determine the absolute photosensitivity.

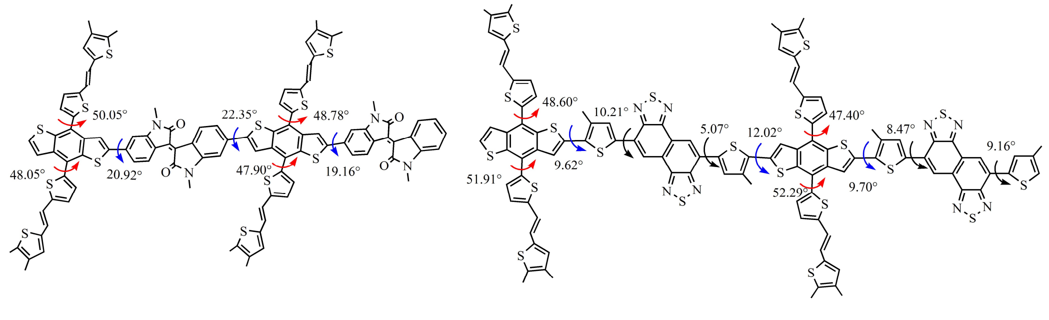

Figure S11. Dihedral angles for model compound $(BDT\text{-}TVT\text{-}ID)_2$ and $(BDT\text{-}TVT\text{-}DTNT)_2$.

Reference

1. Zhao, C.; Zhang, Y.; Ng, M.K. Derivatives of 4,9-dihydro-s-indaceno[1,2-b:5,6-b']dithiop- hene-4,9-dione: Synthesis and Properties. *Org. Chem.* **2007**; *72*, 6364-6371.
